# Genome-Wide Study of NOT2_3_5 Protein Subfamily in Cotton and Their Necessity in Resistance to *Verticillium wilt*

**DOI:** 10.3390/ijms22115634

**Published:** 2021-05-26

**Authors:** Pei Zhao, Tengfei Qin, Wei Chen, Xiaohui Sang, Yunlei Zhao, Hongmei Wang

**Affiliations:** 1 State Key Laboratory of Cotton Biology, Institute of Cotton Research, Chinese Academy of Agricultural Sciences, Anyang 455000, China; zhaopei1986@126.com (P.Z.); cotton55@126.com (W.C.); sangxh2006@126.com (X.S.); 2 Henan Collaborative Innovation Center of Modern Biological Breeding, Henan Institute of Sciences and Technology, Xinxiang 453003, China; qintengfeisam@163.com; 3 Zhengzhou Research Base, State Key Laboratory of Cotton Biology, Zhengzhou University, Zhengzhou 450000, China

**Keywords:** NOT2_3_5 domain protein, *Verticillium wilt*, cotton

## Abstract

The Negative on TATA-less (NOT) 2_3_5 domain proteins play key roles in mRNA metabolism and transcription regulation, but few comprehensive studies have focused on this protein family in plants. In our study, a total of 30 *NOT2_3_5* genes were identified in four cotton genomes: *Gossypium. arboretum*, *G. raimondii*, *G. hirsutum* and *G. barbadense.* Phylogenetic analysis showed that all the NOT2_3_5 domain proteins were divided into two classes. The *NOT2_3_5* genes were expanded frequently, and segmental duplication had significant effects in their expansion process. The *cis*-regulatory elements analysis of *NOT2_3_5* promoter regions indicated that NOT2_3_5 domain proteins might participate in plant growth and development processes and responds to exogenous stimuli. Expression patterns demonstrated that all of the *GhNOT2_3_5* genes were expressed in the majority of tissues and fiber development stages, and that these genes were induced by multiple stresses. Quantitative real-time PCR showed that *GbNOT2_3_5* genes were up-regulated in response to verticillium wilt and the silencing of *GbNOT2_3_5-3/8* and *GbNOT2_3_5-4/9* led to more susceptibility to verticillium wilt than controls. Identification and analysis of the NOT2_3_5 protein family will be beneficial for further research on their biological functions.

## 1. Introduction

Negative on TATA-less (NOT) proteins are important constitutive components of the carbon catabolite repression 4 (CCR4)-NOT complex, which exists in all eukaryotes and plays a key role in mRNA metabolism and transcription regulation [1]. The *NOT* genes of yeast were isolated from mutations in which transcription of the *HIS3* gene was increased [2,3]. Collart et al. indicated that mutations of the NOT genes cause up-regulated expression of many genes [4]. To date, several kinds of NOT proteins have been isolated from yeasts, flies, humans and *Arabidopsis thaliana*, including NOT1, NOT2, NOT3, NOT4, NOT5, NOT9, NOT10 and NOT11 [5]. The NOT proteins function in a complex. Among them, NOT1, as a >200 kDa subunit, functions as a scaffold for the CCR4-NOT complex and other components of the CCR4-NOT complex dock on the NOT1 protein [6]. As core proteins, NOT2, NOT3 and NOT4 docked on the C-terminal domain of the NOT1 scaffold [7,8], while NOT10 and NOT11 dock onto the N-terminal domain of the NOT1 scaffold [9,10].

Genetic and biochemical analysis indicated that NOT2, NOT3 and NOT5 shared a conserved NOT2_3_5 domain and were closely associated [11]. Additionally, *NOT3* and *NOT5* from *Saccharomyces cerevisiae* were regarded as paralogous genes recently [12], and NOT5 had up to 44% sequence identity with NOT3 protein in the N-terminal region of [13]. Many previous studies indicated that NOT2, NOT3 and NOT5 function together and play key roles in the vegetative growth and transcription machinery of yeast [12]. Until now, there have been more studies on NOT proteins containing a NOT2_3_5 domain than on other NOT proteins. Russell et al. identified four *not2* mutations of *Saccharomyces cerevisiae* and suggested that there was no 1.9 MDa CCR4-NOT complex in the *not2:L9* mutant, and stability of the CCR4-NOT complex was decreased slightly in the *not2-4* mutant [14]. Additionally, NOT2 protein had a significant influence on the integrity of the CCR4-NOT complex and the associations with other NOT proteins. Proteins NOT2 and NOT3/5 were also essential for assembly of the proteasome, and together with some chaperones and proteasomes strongly regulated protein synthesis and degradation in the cell [12]. Additionally, NOT2 was found to be involved in transcription elongation and mutation of NOT2 decreased the interaction between RNA polymerase II and NOT proteins [15]. Sohn et al. [16] found that CNOT2 facilitated the differentiation and lipogenesis of 3T3-L1 adipocyte through regulating expression of PPARγ, CEBPα, GSK3α/β and β-catenin. The expression level of CNOT2 was positively correlated with the expression level of PPARγ and CEBPα and negatively correlated with that of GSK3α/β and β-catenin [16]. Besides studies on NOT2_3_5 proteins from yeast and human, there has been some research focused on NOT2_3_5 proteins from phytopathogenic fungi and plants. Gene *FonNot2* was expressed higher in conidia and germinating conidia than in mycelia and played important roles in vegetative growth and conidia production and the conidial morphology of watermelon fusarium wilt pathogen (*Fusarium oxysporum f.* sp.) [17]. The *FonNot2* was significantly up-regulated during infection of watermelon roots compared to mycelia under axenic conditions and FonNot2 increased virulence in the infection process through regulating cell wall integrity, oxidative stress response, ROS production and fatty acids (FA) biosynthesis [17]. In previous studies, two highly similar NOT2 homologs in Arabidopsis were identified including AtNOT2a and AtNOT2b; AtNOT2b was previously known as VIRE2-INTERACTING PROTEIN2 (VIP2) and was isolated using a yeast two-hybrid system. Anand et al. [18] suggested that AtVIP2 contains a NOT domain, which is conserved between plants and animals and that AtVIP2, likely as a transcriptional regulator, was involved in *Agrobacterium*-mediated stable transformation through promoting the integration of T-DNA. Wang et al. [19] reported that NOT2 proteins were vital for plant development and inactivation of Os-NOT2 in rice and At-NOT2 in *Arabidopsis thaliana* caused seedling death and defect in male gametogenesis, respectively. Protein NOT2 interacted with RNA polymerase II and several microRNA (miRNA) biogenesis factors containing DCL1, CBP20, CBP80 and SE and acted as general factors to regulate the level of pri-miRNAs and mature miRNAs involved in miRNA biogenesis. Zhao et al. cloned the *TaVIP2* gene, which encoded a NOT2 protein from wheat and indicated that overexpression of Ta*NOT2* in tobacco increased the efficiency of *Agrobacterium*-mediated transformation and resistance to powdery mildew of tobacco [20].

Cotton is one of the most important economic crops in the world because its fibers are the main natural source for textiles [21]. With the deterioration of global climate, cotton cultivation faces a number of biotic and abiotic stresses [22]. The genomes of diploid and allotetraploid cotton including *Gossypium arboretum*, *G. raimondii*, *G. hirsutum* and *G. barbadense* have been successfully sequenced and released, which helps in excavating functional genes and genome-wide analysis of gene families [23,24]. Although the structure and function of the NOT2_3_5 proteins have been studied in yeasts, human, phytopathogenic fungi and some model organisms, little is known about the NOT2_3_5 protein in cotton. Previous studies indicated that NOT2 played a key role in miRNA biogenesis and miRNA regulated expression level of target genes involving in plant growth, development and response to abiotic and biotic stresses [18]. Thus, we wanted to research the NOT2_3_5 family and attempt to utilize the transcriptional regulation of NOT2_3_5 proteins to increase resistance in cotton.

Here, we characterize NOT2_3_5 domain proteins from diploid and tetraploid cotton species and examine their expression patterns during normal growth and development and after abiotic and biotic stresses. Two of these genes were induced in a verticillium-resistant cotton variety after pathogen inoculation. Verticillium wilt, a soil-borne vascular disease, causes devastating losses of cotton yield and quality every year [25,26].The results will provide new approaches to improve resistance to verticillium wilt in cotton and provide new inspiration on further research on transcriptional regulation of NOT proteins.

## 2. Results

### 2.1. Identification of NOT2_3_5 Domain Protein Family in Cotton

In order to determine the NOT2_3_5 domain protein family in cotton, we conducted an HMMER alignment search on the protein sequences of the genomes of the four cotton species, with the hidden Markov model profile of the NOT2_3_5 domain (PF04153). After filtration of proteins without the NOT2_3_5 domain and non-full-length segments, a total of 5, 5, 10 and 10 NOT2_3_5 domain protein genes were identified in *G. arboretum*, *G. raimondii*, *G. hirsutum* and *G. barbadense*, respectively. All the above genes were numbered consecutively from *GaNOT2_3_5-1* to *GaNOT2_3_5-5*, *GrNOT2_3_5-1* to *GrNOT2_3_5-5*, *GhNOT2_3_5-1* to *GhNOT2_3_5-10* and *GbNOT2_3_5-1* to *GbNOT2_3_5-11*, respectively, according to their localization on chromosomes (Table S1). The protein length of obtained NOT2_3_5 domain proteins ranged within 563-1048 amino acids and mean length was 737 amino acids. The MW and pI were also predicted, with ranges of 59019.22 to 115638.5 kDa and 5.27–9.45, respectively, and corresponding means of 81715.43 kDa and 6.75, respectively. Subcellular localization prediction results suggested all of the NOT2_3_5 domain proteins were predicted to be located in the nucleus (Table S1).

### 2.2. Phylogenetic and Conserved Amino Residue Analysis of NOT2_3_5 Domain Proteins

**Figure 1:**
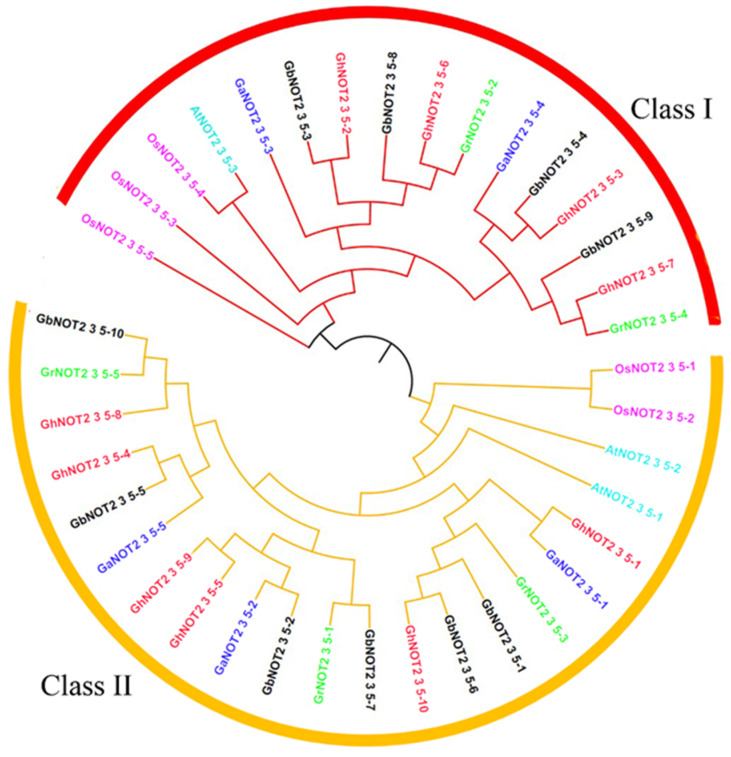
Phylogenetic analysis of NOT2_3_5 genes from cotton and other plants. Prefixes At, Os, Gr, Ga, Gh and Gb represented *Arabidopsis thaliana*, *Oryza sativa*, *G. raimondii*, *G. arboretum*, *G. hirsutum and G. barbadense*, respectively. NOT 2_3_5 domain protein from *G. hirsutum*, *G. barbadense*, *G. raimondii*, *G. arboretum*, *Arabidopsis thaliana* and *Oryza sativa* were marked red, black, green, blue, turquoise and pink, respectively.

To analyze the phylogenetic relationship of NOT2_3_5 domain proteins from four cotton and other plant species, we also obtained three and five NOT2_3_5 domain proteins from *Arabidopsis* and rice, respectively, using the same method. All of the 38 NOT2_3_5 domain protein sequences were aligned using Clustal X. The alignment results were used to construct a phylogenetic tree by MEGA7 software using the NJ method with a bootstrap value set as 500 (Figure 1). All of the NOT2_3_5 domain proteins were divided into two classes: Class I and Class II, with 16 and 22 members. Using the WebLogo online software, the conserved domain-sequence analysis of the two classes of NOT2_3_5 domain proteins was performed. The conserved NOT2_3_5 domains were likely located in the C-terminal and the conservation degrees of amino acid residue from each class of NOT2_3_5 domains were very high (Figure S1). Several amino acid residues showed no variations. However, there were significant differences in the sequences of NOT2_3_5 domains from Classes I and II (Figure S1).

**Figure 2:**
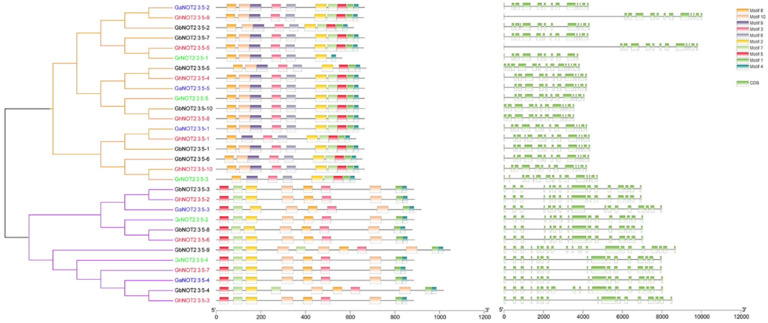
Analysis of conserved motif and gene structure of NOT2_3_5 domain proteins. NOT2_3_5 domain protein from *G. hirsutum*, *G. barbadense*, *G. raimondii* and *G. arboretum* were marked red, black, green and blue, respectively.

Structures of NOT2_3_5 domain protein genes were analyzed by aligning the genomic DNA sequences and coding sequences using the GSDS online software. Genes from the same class had similar gene structures with conserved size and number of exons and variable length of introns. The exon number of NOT2_3_5 domain protein genes from Class I ranged within 10–15. Among them, GrNOT2_3_5-1 and GrNOT2_3_5-3 had the lowest and highest number of exons, respectively. Most NOT2_3_5 domain protein genes from Class II had 16 exons, but GaNOT2_3_5-3 had 17, GbNOT2_3_5-4 had 21 and GbNOT2_3_5-4 had 19. The conserved motifs of the 30 NOT2_3_5 domain protein sequences were predicted by MEME software. The NOT2_3_5 domain proteins from Class I had 7–10 conserved motifs and most had 10. Most NOT2_3_5 domain proteins from Class II contained nine motifs except for GhNOT2_3_5-6 and GbNOT2_3_5-4 with 11 and 9, respectively (Figure 2). All the conserved motif sequences were uploaded to InterPro online tools to analyze their functions. Motifs 1 2, 3, 5, 6, 7, 9 and 10 were classified into NOT2/NOT3/NOT5 protein family membership (IPR040168). Motifs 4 and 8 were not classified into any membership (Table S2).

### 2.3. Chromosome Location and Gene Collinearity Analysis

All NOT2_3_5 domain protein genes from the four cotton species were mapped onto chromosomes or scaffolds. Of 30 NOT2_3_5 domain protein genes, 28 were located on chromosomes and 2 were mapped to scaffolds (Figure S2). Five *GrNOT2_3_5* genes were found in D04, D07, D09 and D13; and five *GaNOT2_3_5* genes were mapped to A05, A08, A11 and A13. Ten *GbNOT2_3_5* genes were mapped to AD2_A05, AD2_A08, AD2_A11 and AD2_A13 and the corresponding D genome. Two of *GhNOT2_3_5* genes were mapped to scaffolds, and the chromosome locations of the remaining *GhNOT2_3_5* genes were similar to those of GbNOT2_3_5 genes. Loci of *GhNOT2_3_5* and *GbNOT2_3_5* genes were deeply conserved between At and Dt sub-genomes in allotetraploid cotton, indicating that *G. hirsutum* and *G. barbadense* were typical allotetraploid species and were applicable to natural polyploidization study.

**Figure 3:**
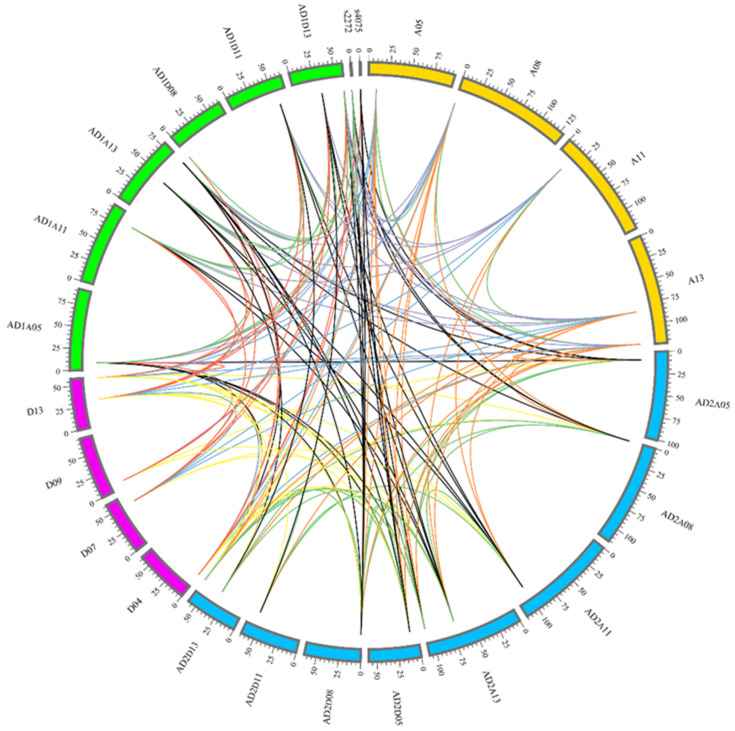
Gene collinearity analysis among cotton *NOT2_3_5* genes. AD1AX and AD1DX (green blocks) indicated chromosomes of A sub-genome and D sub-genome of *G. hirsutum*. AD2AX and AD2DX (blue blocks) indicated chromosomes of A sub-genome and D sub-genome of *G. barbadense*. AX (yellow blocks) and DX (pink blocks) represented chromosomes of *G. arboretum* and *G. raimondii*, respectively. Green lines exhibited duplicated gene pairs among *NOT2_3_5* genes in each *G. arboretum*, *G. barbadense*, *G. hirsutum*, *G. raimondii* cotton species. Orange lines, blue lines, red lines, yellow lines, purple lines and black lines separately exhibited duplicated gene pairs among *NOT2_3_5* genes from and *G. arboretum* and *G. barbadense*, *Gossypium arboretum* and *G. raimondii*, *G. hirsutum* and *G. raimondii*, *G. raimondii* and *G. barbadense*, *G. hirsutum* and *G. arboretum*, *G. barbadense* and *Gossypium arboretum*.

Gene families are created by duplications and variations from a common ancestral gene. Gene duplication events play key roles in species evolution and environmental adaptation. Segmental duplication and tandem repeat are the main types of gene duplications. MCSCAN software was used to analyze the expansion and synteny of NOT2_3_5 domain protein genes from *G. arboretum*, *G. raimondii*, *G. hirsutum* and *G. barbadense*. The duplication events were selected according to the standard described in the methods. Results indicated that all gene family members of NOT2_3_5 domain proteins were produced by duplication events and segmental duplication events, but no tandem duplication events were observed in their expansion process. There were 155 pairs of duplicated genes of NOT2_3_5 domain proteins identified among the four cotton species. There were 4, 17, 12 and 2 duplicated gene pairs observed in *G. arboretum*, *G. barbadense*, *G. hirsutum* and *G. raimondii,* respectively. There were 19, 11 and 22 pairs of segmental duplication events detected between *G. arboretum* and *G. hirsutum*, *G. arboretum* and *G. raimondii* and *G. arboretum* and *G. barbadense,* respectively. There were 18, 18 and 32 pairs of segmental duplication events obtained between *G. barbadense* and *G. raimondii*, *G. hirsutum* and *G. raimondii* and *G. barbadense* and *G. hirsutum*, respectively (Figure 3). Results suggested that genes of NOT2_3_5 domain proteins were expanded frequently, and segmental duplication had a significant effect on their expansion.

The Ka/Ks ratio was applied to predict the selection pressure on gene pairs in the four cotton species, with Ka/Ks = 1, >1 and <1 representing neutral, positive and purifying selections, respectively. Results suggested a Ka/Ks < 1 for 153 NOT2_3_5 domain protein gene pairs indicating that the duplicated genes in the four species had evolved mainly by purifying selection (Figure S3). Additionally, only one gene pair (*GbNOT2_3_5-3* and *GaNOT2_3_5-3*) processed a Ka/Ks > 1, which suggested that that gene pair experienced positive selection pressure. Detailed information for the Ka/Ks ratio of duplicated gene pairs of NOT2_3_5 domain proteins is given in Table S3.

### 2.4. Cis-Regulatory Elements Analysis of NOT 2_3_5 Promoter Regions

**Figure 4:**
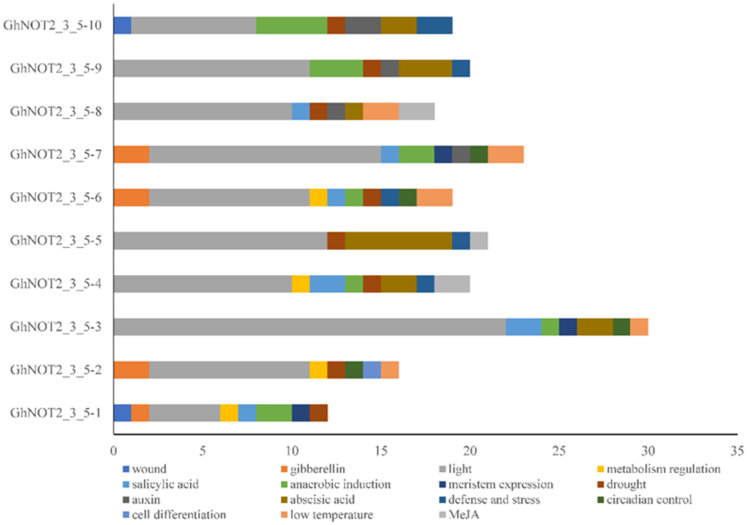
*Cis*-Regulatory elements analysis of *NOT 2_3_5* promoter regions. Different colors represented different *cis*-regulatory elements and numbers in abscissa were the numbers of *cis*-regulatory element.

The *cis*-regulatory elements of promoter regions play vital roles in regulating gene expressions in serious conditions and plant developmental stages. Through identifying and analyzing *cis*-regulatory elements, the expression patterns, regulatory network and potential functions of NOT2_3_5 domain protein genes were studied deeply. The 2.5-kb upstream genomic sequences from the start codon were considered as putative promoter regions and applied to predict *cis*-regulatory elements. There were 198 *cis*-elements (15 types) obtained from 10 putative *GhNOT2_3_5* promoters, which were classified into three groups: Cellular development and metabolism, phytohormone regulation and abiotic and biotic stress response (Figure 4 and Table S4). The *cis*-elements mentioned above, involved in cellular development and metabolism, included O_2_-site, CAT-box, HD-Zip 1, circadian and MBSI, which represented zein metabolism, meristem expression, palisade mesophyll cell differentiation, circadian control and flavonoid biosynthetic gene regulation, respectively. Additionally, more than half of the *cis*-elements (107, 18 different kinds) were associated with light response. In the phytohormone regulation group, five types of *cis*-elements were detected: P-box (gibberellin-responsive element), ABRE (abscisic acid responsiveness), TCA-element (SA responsiveness), CGTCA-motif (MeJA-responsiveness) and AuxRR-core (auxin-responsive element). There were five types of *cis*-elements in the abiotic and biotic stress response group: WUN-motif, MBS, ARE, TC-rich repeats and LTR, which responded to wound, drought, anaerobic environment, pathogenic bacteria and low temperature, respectively. All the results indicated that NOT2_3_5 domain proteins might participate in plant growth and development processes and respond to exogenous stimuli.

### 2.5. Prediction of MiRNAs Targeting GhNOT2_3_5 Domain Protein Genes

**Figure 5:**
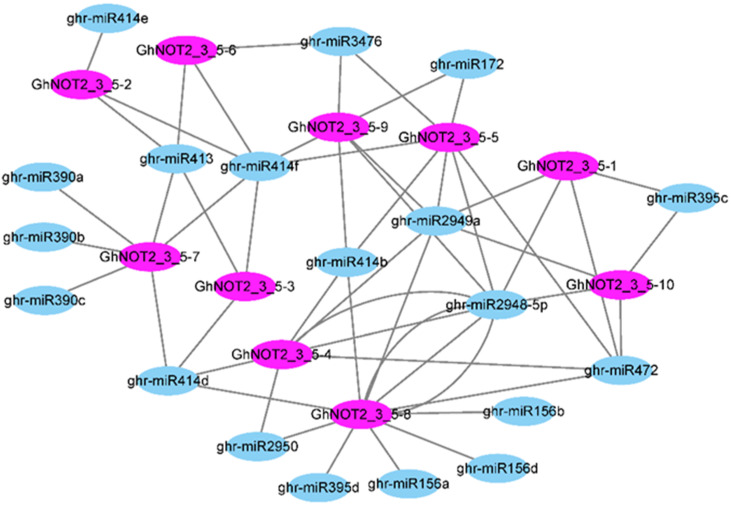
Prediction of miRNAs targeting *GhNOT2_3_5* genes. Connections among nodes and lines indicate the targeting relationship of miRNA and *GhNOT2_3_5* genes.

Plant regulatory small RNAs, especially miRNAs, play key roles in regulating gene expression and are involved in plant responses to abiotic and biotic stress processes. The prediction of miRNAs targeting genes would shed new light on understanding the crosstalk between miRNAs from upland cotton and NOT2_3_5 domain protein genes. There were 55 interaction relationships obtained, including 19 ghr-miRNAs targeting 10 *GhNOT2_3_5* genes (Figure 5). Among them, ghr-miR2949a targeted six *GhNOT2_3_5* genes (*GhNOT2_3_5-1*, *GhNOT2_3_5-4*, *GhNOT2_3_5-5*, *GhNOT2_3_5-8*, *GhNOT2_3_5-9* and *GhNOT2_3_5-10*) indicating that ghr-miR2949a was closely related to the expression regulation of *GhNOT2_3_5* genes. Gene *GhNOT2_3_5-8* was potentially targeted by nine ghr-miRNAs (ghr-miR2950, ghr-miR395d, ghr-miR156a, ghr-miR156d, ghr-miR2948-5p, ghr-miR472, ghr-miR2949a, ghr-miR414b and ghr-miR414d), making it the most targeted *GhNOT2_3_5* gene. Especially, *GhNOT2_3_5-8* was targeted by ghr-miR2948-5p at three different sites. Additionally, other *GhNOT2_3_5* genes were also targeted by several ghr-miRNAs: *GhNOT2_3_5-4*, *GhNOT2_3_5-5*, *GhNOT2_3_5-7* and *GhNOT2_3_5-9* were predicted to be targeted by 5–6 ghr-miRNAs.

### 2.6. Expression Patterns of NOT2_3_5 Genes in Different Tissues and Responding to Stresses and Phytohormones

**Figure 6:**
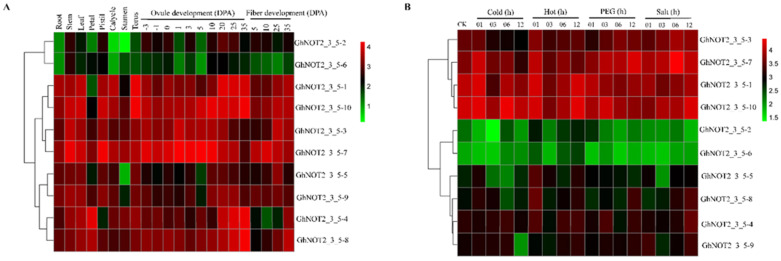
Heat maps of expression patterns of *NOT2_3_5* genes. (**A**) Expression patterns of *NOT2_3_5* genes in different tissues of *G. hirsutum.* (**B**) Expression patterns of *NOT2_3_5* genes under different stresses of *G. hirsutum*.

Gene functions have a close relationship with their expression profiles including expression level and expression location. To better and further study the function of NOT2_3_5 domain protein, the expression profiles of *GhNOT2_3_5* genes in different tissues of root, stem, leaf, petal, pistil, calycle, stamen, torus and in different fiber and ovule development stages 5, 10, 20 and 25 days post-anthesis (DPA) for fiber and -3, -1, 0, 1, 3, 5, 10, 20, 25 and 35 DPA for ovules were analyzed using *G. hirsutum* RNA-seq data [27]. Results demonstrated that all GhNOT2_3_5 genes were expressed in all tissues and development stages mentioned above, but their expression levels varied. However, the expression patterns of *GhNOT2_3_5* genes from the A sub-genome were similar to genes from the corresponding D sub-genome. We observed that *GhNOT2_3_5-1-3/7* were abundantly expressed with mean values of log_2_ FPKM > 3.5, while *GhNOT2_3_5-1-2/6* were slightly expressed with values of log_2_ FPKM < 2.0, suggesting that duplicated *GhNOT2_3_5* genes might have diverse biological functions. Genes *GhNOT2_3_5-1-7* and *GhNOT2_3_5-8* were up-regulated in petal, pistil, calycle, stamen and torus, indicating significant roles in floral development. Gene *GhNOT2_3_5-10* showed a continuous increase in expression level during fiber and ovule development processes. Gene *GhNOT2_3_5-1* had higher expression levels in root, stem and leaf than other tissues suggesting a function associated with stress resistance. The expression profile of GhNOT2_3_5 genes indicated that the NOT2_3_5 domain protein family might be involved in all cotton growth processes and in fiber and ovule development (Figure 6A). Meanwhile, the expression profiles of *GhNOT2_3_5* genes responding to stresses including cold, hot, polyethene glycol (PEG) and salt were also investigated using the RNA-seq data. The heat map suggested that *GhNOT2_3_5-1/*10/3/7 genes were expressed highly during all stress treatments but *GhNOT2_3_5-2*/6 genes were barely expressed. Under hot treatment, *GhNOT2_3_5-5* were up-regulated at an early treatment stage compared to the control. The expression level of *GhNOT2_3_5-7* was induced by PEG and salt treatment, with peak expression at 12 h after PEG treatment and 6 h after salt treatment. Gene *GhNOT2_3_5-9* was down-regulated after 12h of cold treatment (Figure 6B).

**Figure 7:**
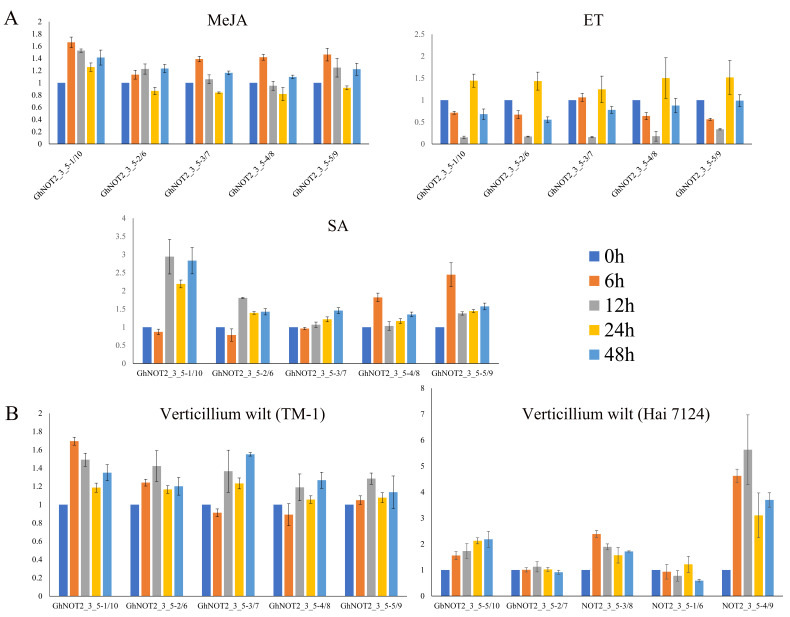
Expression patterns of *NOT2_3_5* genes under exogenous hormone and after infection of *V. dahlia* using qPCR. (**A**) Expression patterns of *NOT2_3_5* genes in *G. hirsutum* var TM-1 under exogenous hormone, including SA, MeJA and ET, treatment. (**B**) Expression patterns of *NOT2_3_5* genes in *G. barbadense* var Hai 7124 after infection of *V. dahlia*.

The predicted results of *cis*-regulatory elements from *GhNOT2_3_5* putative promoter regions suggested that *GhNOT2_3_5* genes might respond to exogenous hormones and pathogenic bacteria. Thus, expression patterns of *GhNOT2_3_5* members in *G. hirsutum* var TM-1 and *G. barbadense* var Hai 7124 under exogenous hormone treatment (SA, MeJA and ET), t and after *V. dahliae i*nfection were analyzed using qPCR. The expression levels of *GhNOT2_3_5* genes did not significantly change under MeJA treatment indicating that they were not involved in MeJA response pathways. All of the *GhNOT2_3_5* genes were down-regulated at 12h after ET treatment. However, gene *GhNOT2_3_5-1/10* was slightly down-regulated at 6h after treatment and up-regulated after experiencing a long period of SA treatment. Gene *GhNOT2_3_5-5/9* showed a higher expression level at the early SA treatment time point (Figure 7A). Expression of each gene pair between 0 time point for each exogenous hormones treatment experiment did not vary more than 1.339 fold. Genes of *NOT2_3_5* from *G. hirsutum* and *G. barbadense* exhibited different expression patterns following *V. dahlia* infection. None of the *GhNOT2_3_5* genes were induced by *V. dahliae* infection, suggesting that they had no roles in cotton resistance against verticillium wilt. Notably, expression levels of *GbNOT2_3_5-5/10*, *GbNOT2_3_5-3/8* and *GbNOT2_3_5-4/9* were up-regulated under *V. dahliae* treatment. Among them, *GbNOT2_3_5-4/9* showed significantly higher expression at four treatment time points, with expression peaking at 12 h after inoculation. The expression level of *GbNOT2_3_5-5/10* gene increased during the inoculation process. Gene *GbNOT2_3_5-3/8* was induced early, with expression peaking at 6 h after inoculation. The expression profiles of *NOT2_3_5* under *V. dahliae* inoculation was in accordance with disease resistance of cotton varieties. The *G. barbadense* var Hai 7124 is *V. dahliae* resistant and some *GbNOT2_3_5* genes were up-regulated during *V. dahliae* inoculation; however, *G. hirsutum* var TM-1 is V. *dahliae* susceptible and all of its *GhNOT2_3_5* genes were not induced under treatment (Figure 7B).

### 2.7. Silencing of GbNOT2_3_5 Genes Reduced Verticillium Wilt Resistance in Cotton

**Figure 8:**
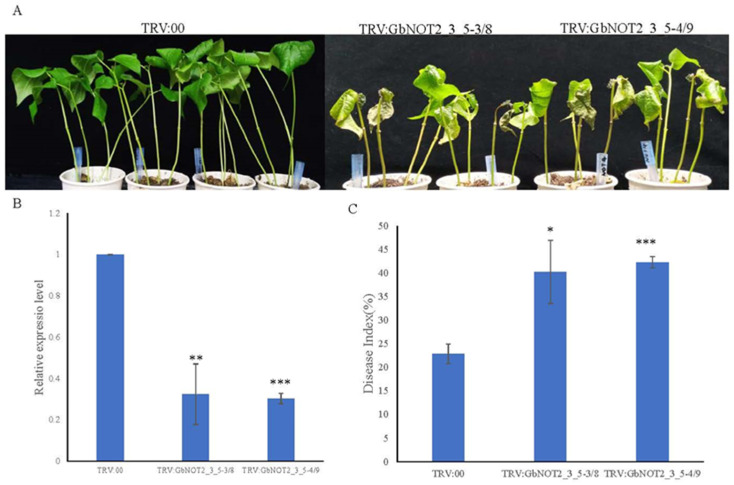
Silencing *GbNOT2_3_5* genes reduced Verticillium wilt resistance in cotton. (**A**) The phenotypes of silencing plants and control after infection of *V. dahlia*. TRV:00 represented empty vector, TRV:GbNOT2_3_5 represented *GbNOT2_3_5* genes silenced. (**B**) Expression levels of *GbNOT2_3_5* genes in silencing plants and control. (**C**) The DI values of silencing plants and control after infection of *V. dahlia*. * *p* < 0.1 by Student ’s *t* test, ** *p* < 0.05 and *** *p* < 0.01.

Tobacco rattle virus (TRV)-based VIGS systems were used to further study whether *GbNOT2_3_5* genes played roles in cotton responses to verticillium wilt. Genes *GbNOT2_3_5-3/8* and *GbNOT2_3_5-4/9* were selected because their expression levels were significantly up-regulated during *V. dahliae* inoculation. About 300 bp of *GbNOT2_3_5-3/8* and *GbNOT2_3_5-4/9* genes were amplified using suitable primers and were inserted into the pTRV2 vector, forming recombinant vector pTRV2–*GbNOT2_3_5-3/8* and pTRV2–*GbNOT2_3_5-4/9*. An *Agrobacterium* strain carrying the above recombinant plasmids was injected into cotyledons. After VIGS, the expression level of *GbNOT2_3_5-3/8* and *GbNOT2_3_5-4/9* genes were significantly reduced (Figure 8B). After the TRV:PDS cotton seedlings showed a photobleaching phenotype across all true leaves, the remaining cotton seedlings infiltrated with recombinant vectors and empty vectors were inoculated with *V. dahliae*. After 25 d, *GbNOT2_3_5* silenced cotton displayed obvious symptoms of verticillium wilt compared to the control, including wilting of true leaves and yellowing and falling cotyledons and true leaves (Figure 8A). The DI values of TRV: *GbNOT2_3_5-3/8*, TRV: *GbNOT2_3_5-4/9* were remarkably higher than of the control (TRV:00) after inoculation, with values of 40.27 and 42.32 versus 22.90, respectively (Figure 8C). Stems of silenced cotton seedlings and controls were extracted and cut through the middle of the vascular bundle. The xylem of TRV: *GbNOT2_3_5* plants showed significantly more browning than TRV:00 plants (Figure S4). Trypan blue assay showed that leaves from silenced cotton were severely dyed blue, indicating more dead cells in the leaves of TRV:*GbNOT2_3_5* plants after *V. dahliae* infection (Figure S4). All of the above results showed that silencing *GbNOT2_3_5-3/8* and *GbNOT2_3_5-4/9* genes compromised cotton resistance during *V. dahliae* inoculation.

## 3. Discussion

The NOT2_3_5 domain proteins are important constitutive components of the CCR4-NOT complex and are evolutionarily conserved proteins in eukaryotes. Research on NOT2_3_5 domain proteins has mainly focused on yeast and mammals and those in plants have not been well studied. To further examine NOT2_3_5 domain proteins, we comprehensively analyzed the NOT2_3_5 protein subfamily in four diploid and allotetraploid cotton species. The structural, conserved domain, evolution direction, chromosome location and expression features of the NOT2_3_5 domain proteins were characterized.

### 3.1. Evolution and Duplication of NOT2_3_5 Genes

During evolution, whole-genome duplication (WGD)/polyploidization has occurred in numerous organisms, accompanied by gene movement, gene loss, chromosome structural changes and gene functional divergence [28]. Thus, WGD is one of the main driving forces of genome evolution and formation of new species [29]. Cotton is an ideal model for exploring the mechanism of natural polyploidy, and understanding polyploid evolution in cotton might improve our insight concerning other polyploid plants [30]. The Gossypium contains about 45 diploid species grouped into eight monophyletic groups: A, B, C, D, E, F, G and K genomes. According to their locations, diploid cotton species are divided into three main lineages: the C, G and K genomes (*Sturita* subgenus) derived from Australia; the D genome (*Houzingenia* subgenus) originated from America; and the A, B, E and F genomes derived from Africa and Asia [30]. The formation of the allotetraploid cotton species occurred 1–2 million years ago and the closest extant progenitors of tetraploid cotton are *G. herbaceum* L.(A1) and *G. raimondii* Ulbrich (D5) [31,32]. Because of polyploidization and the formation of allotetraploid cotton species, the number of NOT2_3_5 family members from allotetraploid cotton species is double that in diploid species. Meanwhile, WGD resulted in expansion of gene families of cotton species and NOT2_3_5 domain protein genes were duplicated remarkably. The results of collinearity analysis indicated that segmental duplication was the main approach during duplication of the *NOT2_3_5* gene family. In tetraploid cotton species, the chromosome location of some of NOT2_3_5 family members did not correspond with that in diploid cotton species, indicating that during evolution of the *NOT2_3_5* gene family, chromosome structural changes and chromosome rearrangement may have occurred. Genes of NOT2_3_5 domain proteins in the four cotton species mainly experienced purifying selection, suggesting that differentiation of gene function was partly inhibited.

### 3.2. Relationship between MiRNA and NOT2_3_5 Genes

As a class of non-coding RNA, miRNA are the key molecular device of RNA silencing, which is involved in regulating gene expression at either the posttranscriptional or transcriptional levels. The miRNAs regulate expression levels of target genes involved in plant growth, development and response to abiotic and biotic stresses [33]. Previous studies have shown that the *AtNOT2b* protein is involved in regulating the level of pri-miRNAs and mature miRNAs via interacting with RNA polymerase II and several miRNA biogenesis factors containing DCL1, CBP20, CBP80 and SE. In our study, the prediction of miRNAs targeting GhNOT2_3_5 domain protein genes produced 55 interaction relationships including 19 ghr-miRNAs targeting 10 *GhNOT2_3_5* genes, which indicated that NOT2_3_5 domain proteins had a close relationship with miRNAs. The NOT2_3_5 domain protein regulated the biogenesis of miRNA and, in turn, the expression level of *NOT2_3_5* genes was regulated by some miRNAs. Previous studies have shown that many plant miRNAs are involved in stress response for example miR414 and miR390 are involved in salinity stress [34,35]; miR471, miR472 and miR395 are involved in immune response [36]; miR156 is involved in drought stress [37] and miR2949 is involved in high temperature response [38]. Thus, the prediction results of miRNAs targeting *GhNOT2_3_5* genes inspired us to study the mechanism underlying *GhNOT2_3_5* genes responding to various stresses in depth.

### 3.3. Differences between NOT2_3_5 Domain Proteins from Two Classes

Phylogenetic analysis showed that all of the NOT2_3_5 domain proteins were divided into two classes: Class I and Class II. There were significant differences among the NOT2_3_5 domain proteins from different classes, including: sequences, gene structure and conserved motifs. However, the biological functions of NOT2_3_5 domain proteins were uncertain in cotton. In our study, we found three pairs of the *GbNOT2_3_5* gene, referred to as *GbNOT2_3_5-3/8* and *GbNOT2_3_5-4/9*, were significantly up-regulated after inoculation with *V. dahliae*. Another three gene pairs of *GbNOT2_3_5* gene did not remarkably respond to Verticillium wilt. Gene pairs of *GbNOT2_3_5-3/8* and *GbNOT2_3_5-4/9* were from Class I and another gene pair was from Class II. Thus, these previously mentioned differences between different classes partly resulted in the function division among the protein family. The *cis*-regulatory elements of promoter regions among *GhNOT2_3_5* members were analyzed, and response elements related to light, environmental stress, plant hormones and metabolism were detected. The distribution and kind of *cis*-regulatory elements among *GhNOT2_3_5* members from the same class were discriminatory. Because cis-regulatory DNA sequences of promoter region were the key factors for controlling the accurate spatiotemporal gene expression process, this was essential for plant development and response to external stimulus [39]. Various *cis*-regulatory elements in promoter regions promote the diversification of genes function and make plants more adaptable to environmental changes.

### 3.4. Expression Patterns and Putative Disease Resistant Functions of NOT2_3_5 Domain Protein Genes

Expression patterns of *GhNOT2_3_5* genes in various tissues were studied and showed that all *GhNOT2_3_5* genes were expressed in all tissues and fiber development stages, suggesting that they were involved in the whole cotton growth process and that NOT2_3_5 proteins were important constitutive components of the CCR4-NOT complex and played critical roles in transcription regulation. Similarly, expression profiles of *GhNOT2_3_5* genes under abiotic and biotic stresses were investigated. It is well known that abiotic and biotic stresses including verticillium wilt, drought, salt and heat seriously restrict cotton production all over the world. However, few studies have focused on NOT2_3_5 domain proteins involved in plant response to abiotic and biotic stresses. In our study, some NOT2_3_5 domain proteins were differentially expressed under various stresses and might demonstrate that *GhNOT2_3_5* genes were involved in responding to exogenous stimulus. Especially, some *GbNOT2_3_5* genes (including *GbNOT2_3_5-5/10*, *GbNOT2_3_5-3/8* and *GbNOT2_3_5-4/9)* were significantly up-regulated after infection of verticillium wilt. To further study the function of GbNOT2_3_5 genes in resistance to verticillium wilt, silencing of *GbNOT2_3_5* genes using VIGS technology compromised resistance to *V. dahliae* in *G. barbadense* var Hai 7124. Moreover, the *cis*-regulatory elements analysis of promoter regions suggested that several *NOT2_3_5* genes contained a TCA element (SA responsiveness) and were up-regulated under SA treatment. There is growing evidence that SA is involved in plant disease resistance and regulates expression of pathogenesis-related protein [40]. Thus, we speculate that *GbNOT2_3_5-3/8* and *GbNOT2_3_5-4/9* genes participated in the SA signal pathway and enhanced resistance to verticillium wilt in cotton.

## 4. Materials and Methods

### 4.1. Identification of the NOT2_3_5 Domain Protein Family

The genome sequences of four cotton species were downloaded from the CottonFGD website (https://cottonfgd.org/ (accessed on 14 November 2019)): *G. arboretum* (CRI), *G. raimondii* (JGI), *G. hirsutum* (NAU) and *G. barbadense* (NAU). [27]. The genome sequences of *Arabidopsis*, *Brachypodium sylvaticum* and rice were downloaded from the JGI website (https://phytozome.jgi.doe.gov/pz/portal.html (accessed on 14 November 2019)). The Pfam protein family databases with the NOT2_3_5 domain (PF04153) were used with HMMER software version 3.0 to identify the NOT2_3_5 domain protein in these four cotton and other plant species mentioned above (E < 10^−10^) [41]. The obtained protein sequences were further verified using the SMART database [42]. After selection, sequences with no NOT2_3_5 domain were deleted and the remaining NOT2_3_5 domain protein sequences were recognized as belonging to NOT2_3_5 domain protein family in cotton and other plant species. The ExPASy database (http://www.expasy.org/ (accessed on 8 December 2019)) was used to analyze the protein sequence length, molecular weight and isoelectric point (pI) [43]. The CELLO v2.5 server was applied in predicting the subcellular localization information [44].

### 4.2. Construction of Phylogenetic Tree and Chromosome Map

Clustal X software was used to perform the multiple sequence alignments of NOT2_3_5 domain proteins from four cotton and other plant species [45]. The phylogenetic tree was constructed by MEGA7 software using the neighbor-joining (NJ) method with bootstrap value set as 500 [46]. MapChart 2.2 software was used to analyze the respective chromosome distributions of genes encoding the NOT2_3_5 domain proteins in four cotton species [47].

### 4.3. Gene Collinearity and Structural Analysis

The protein sequences of four cotton species were aligned with each other using the Basic Local Alignment Search Tool (BLAST). The collinearity analysis was performed using MCscan software according to the above blast results. Collinear pairs in the NOT2_3_5 domain protein family were selected, and CIRCS software was used to draw the collinearity map [48]. Additionally, to analyze the divergence of NOT2_3_5 families, their coding sequences were aligned using MEGA6.0 software and the non-synonymous substitution rate (*Ka*) and synonymous substitution rate (*Ks*) values of the orthologous gene pairs were calculated using DnaSP5.0 software. The *Ka*/*Ks* ratio was applied to estimate the selection pressure for the duplication events of NOT2_3_5 families. The exon and intron positions were obtained by aligning the genomic DNA sequences and coding sequences using the Gene Structure Display Server (GSDS) online software. The Multiple Em for Motif Elicitation (MEME) software was used to analyze the protein sequences from four cotton species.

### 4.4. Target Prediction between MiRNAs and GhNOT2_3_5 Members

The miRNA sequences of upland cotton (*G. hirsutum*) were downloaded from the plant microRNA database. The transcript sequences of *GhNOT2_3_5* families were uploaded to the psRNATarget server (2017 update) and predicted candidate target transcripts against *G. hirsutum* miRNA sequences [49]. The target prediction results of *G. hirsutum* miRNA and *GhNOT2_3_5* families were displayed using Cytoscape software [50].

### 4.5. Prediction of Upstream cis-Elements

The 2.5-kb upstream genomic sequences from the start codon of each *NOT2_3_5* gene were extracted from the genome files of the four cotton species. The above-2.5-kb promoter regions were submitted to the Plant CARE database (http://bioinformatics.psb.ugent.be/webtools/plantcare/ (accessed on 13 Feburary 2020)) to predict *cis*-elements of NOT2_3_5 gene promoters [51].

### 4.6. Expression Pattern Analysis

The expression patterns of *GhNOT2_3_5* members in *G. hirsutum* var TM-1 in various tissues and under different stresses were analyzed using the RNA-seq data, which were downloaded from the CottonFGD website (https://cottonfgd.org/(accessed on 15 May 2020)) [27]. The heat maps were generated using the log_2_FPKM values by MeV software. Expression patterns of *GhNOT2_3_5* members in *G. hirsutum* var TM-1 under exogenous hormone treatment and after infection of verticillium wilt were analyzed using quantitative real-time PCR (qPCR) data. Selected primer pairs were predicted to be specific to each gene pair based on the sequence alignment. Seedlings of *G. hirsutum* var TM-1 were grown in plant growth chambers at 25 °C during the day and 23 °C at night, 16/8 h light/dark photoperiod, and 70% relative humidity. For the exogenous hormone treatment, exogenous hormones: salicylic (SA), ethylene (ET) and methyl jasmonate (MeJA) were sprayed onto leaves at 1 μmol/mL when the cotton seedlings possessed two true leaves. Each time point represents RNA isolated from the leaves of 4 different plants harvested 0, 6, 12, 24, and 48 h after treatment (total of 20 plants/treatment) and then stored at −80 °C. For inoculation of verticillium wilt, roots of cotton seedlings with two true leaves were uprooted gently, dipped in the 10 mL of conidial suspensions of *Verticillium dahliae* (1 × 10^7^ conidia/mL, Vd991) for 5 min and the plants replanted in the pots. Mixed roots, isolated from the roots of 4 different plants, were harvested 0, 6, 12 and 24 h after treatment and then stored at −80 °C. Total RNA of the above plant samples were isolated using RNAprep Pure Plant Plus Kit (Polysaccharides & Polyphenolics-rich) (TIANGEN, Beijing, China) according to the manufacturer’s instructions, and cDNA was synthesized using PrimeScript^TM^ II 1st strand cDNA Synthesis Kit (TaKaRa, Dalian, China). The qPCR was performed on QuantStudio ^TM^ 6 Flex Real-Time PCR System (Applied Biosystems, Carlsbad, CA, USA) using TB Green Premix Ex Taq^TM^ (Tli RNaseH Plus), Bulk (TaKaRa, Dalian, China). Cotton *actin* gene was used as an internal control gene for normalization of expression values. All primers used in this study are listed in Table S5.

### 4.7. Vector Construction and Development of Virus-Induced Gene Silencing (VIGS) in Cotton

About 300 bp of *GbNOT2_3_5-3/8* and *GbNOT2_3_5-4/9* gene fragments were amplified by corresponding primers. The above fragments were inserted into pYL156 vector by ClonExpress^TM^ II One Step Cloning Kit (Vazyme, Nanjing, China). Plasmids containing the pYL156–*GbNOT2_3_5-3/8*, pYL156–*GbNOT2_3_5-4/9*, pYL156–*GhPDS*, pYL156 and pYL192 were transformed into *Agrobacterium tumefaciens* GV3101 by the freeze–thaw method, respectively [52]. The *A. tumefaciens* containing recombinant plasmids were grown overnight at 28 °C and collected. The *A. tumefaciens* carrying pYL156–*GbNOT2_3_5-3/8* and pYL156–*GbNOT2_3_5-4/9* were respectively mixed with *A. tumefaciens* carrying pYL192 in a 1:1 ratio. Mixed *A. tumefaciens* were injected into two fully expanded cotyledons of cotton seedlings using sterile needleless injectors [52]. When the positive control plants appeared as albino phenotypes, expression level of above *GbNOT2_3_5* gene were tested. Inoculation with *V. dahliae* was performed as described above until purposed *GbNOT2_3_5* gene were silenced. VIGS experiments were performed with at least three biological repeats and for each repeat, there were more than 10 plants per constructed vector. Morbidity situation was investigated 25 d after inoculation with *V.dahliae.*

## Data Availability

The genome sequences of plant species and RNA-seq data used in our manuscript were downloaded from the CottonFGD website (https://cottonfgd.org/).

## Supplementary Materials

The following are available online at https://www.mdpi.com/article/10.3390/ijms22115634/s1, Figure: S1: Conserved domain-sequence and amino residues analysis of NOT2_3_5 domain proteins. A: Conserved domain-sequence analysis. NOT2_3_5 domain were located in the C-terminal. B: Conserved amino residues analysis. The conservation degrees of amino acid residue from each class of NOT2_3_5 domains were very high. Figure: S2 Chromosome Location of *NOT2_3_5* genes from four cotton species. Abbreviated A2, D5, AD1, AD2 represented *G. arboretum*, *G. raimondii*, *G. hirsutum* and *G. barbadense*, respectively. Figure S3: Distribution of Ka/Ks ratios of NOT2_3_5 gene pairs in tetraploid cotton. A: Ka/Ks ratios between A and D sub-genomes of *G. hirsutum*. B: Ka/Ks ratios between *G. hirsutum* and *G. barbadense.* C: Ka/Ks ratios between A and D sub-genomes of *G. barbadense*. Circles represented outlier and triangles represented average value. Figure S4: The stems and leaves of silencing *GbNOT2_3_5* genes plants showed more severe symptoms after infection of *V. dahlia*. A: The stems from silencing *GbNOT2_3_5* genes plants and control. B: Trypan blue array of leaves from silencing *GbNOT2_3_5* genes plants and control. Table S1: The detailed information of NOT2_3_5 domain protein family from four cotton species. Table S2: The top ten conserved motifs and the functional searches on InterProScan database. Table S3: The details information for the Ka/Ks ratio of duplicated genes pairs of NOT2_3_5 domain proteins. Table S4: *Cis*-elements from 10 putative GhNOT2_3_5 promoter regions. Table S5: Primer sequences used in the study.

## Abbreviations

NOT	Negative on TATA-less
CCR4-NOT	Carbon Catabolite Repression 4-NOT
SA	Salicylic Acid
ET	Ethylene
MeJA	Methyl Jasmonate
VIGS	Virus-Induced Gene Silencing
pI	isoelectric point
MW	molecular weight
Dpa	Days Post Anthesis
TRV	Tobacco Rattle Virus
