# Supplementary material for: Genome-Wide Study of NOT2_3_5 Protein Subfamily in Cotton and Their Necessity in Resistance to Verticillium wilt
Source: Int J Mol Sci. 2021 May 26;22(11):5634. doi: 10.3390/ijms22115634 (PMC8198034; doi:10.3390/ijms22115634)
Supplement: Supplementary file 1 [file ijms-22-05634-s001.zip › supplementary figures-FINAL-FOR-XML.pdf]

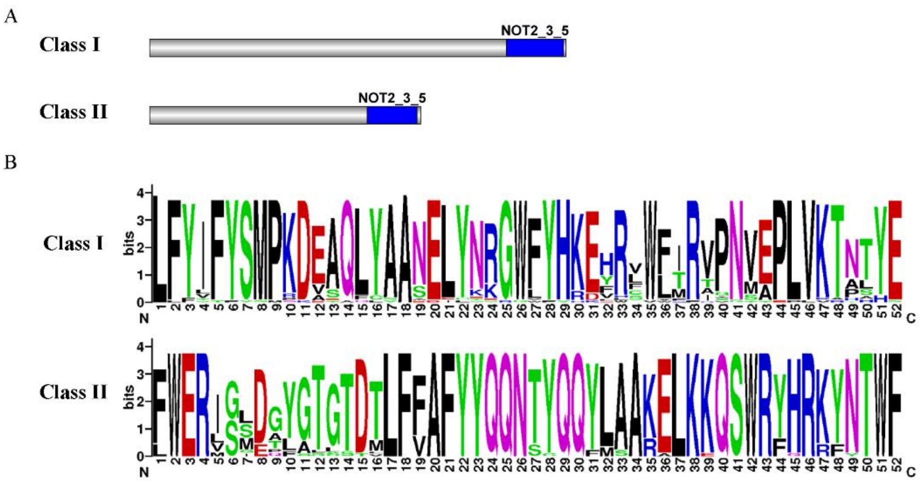

Figure S1: Conserved domain-sequence and amino residues analysis of NOT2_3_5 domain proteins. A: Conserved domain-sequence analysis. NOT2_3_5 domain were located in the C-terminal. B: Conserved amino residues analysis. The conservation degrees of amino acid residue from each class of NOT2_3_5 domains were very high.

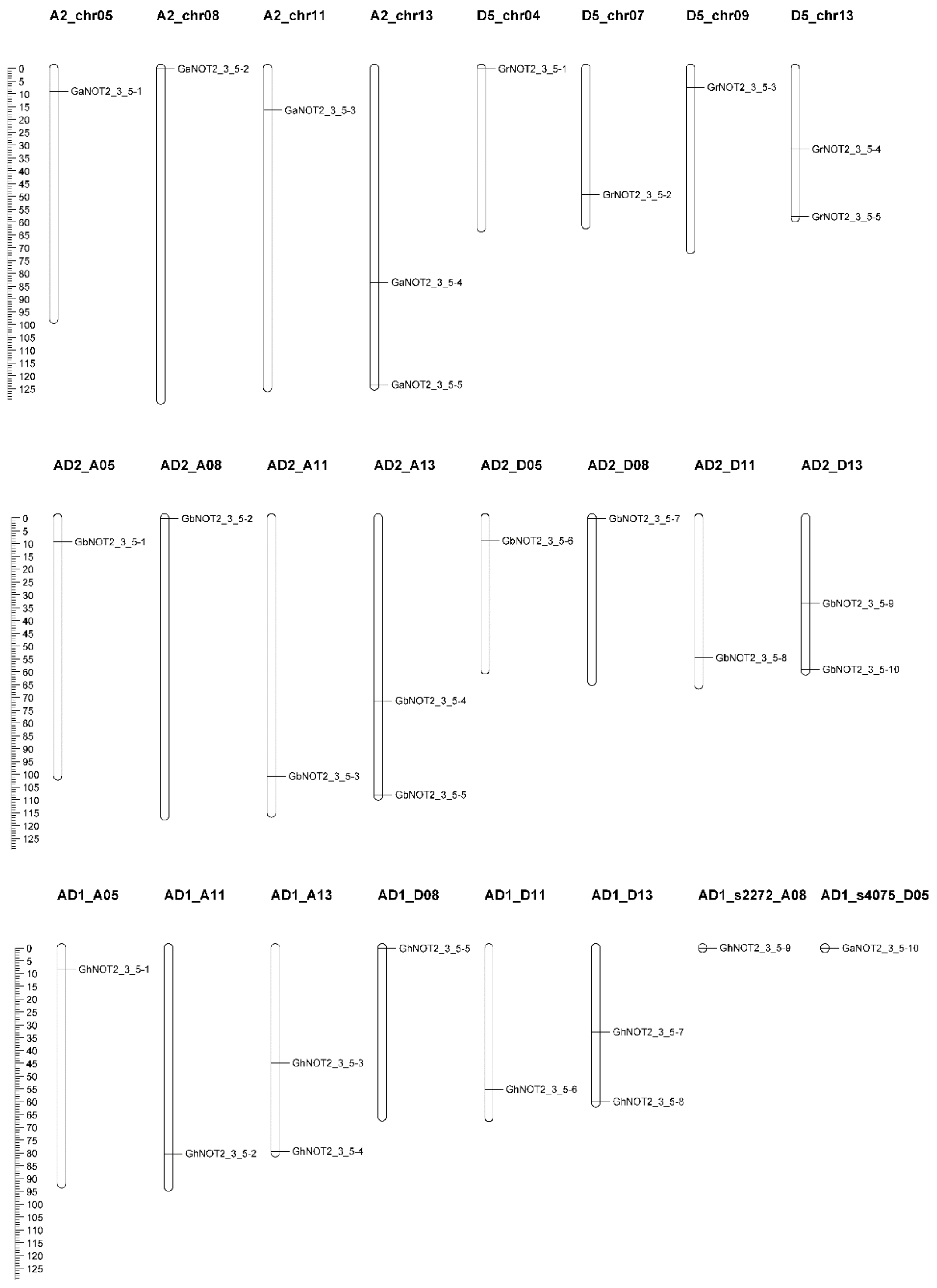

Figure S2: Chromosome Location of *NOT2_3_5* genes from four cotton species.
Abbreviated A2, D5, AD1, AD2 represented *G. arboretum, G. raimondii, G. hirsutum* and *G. barbadens*e, respectively.

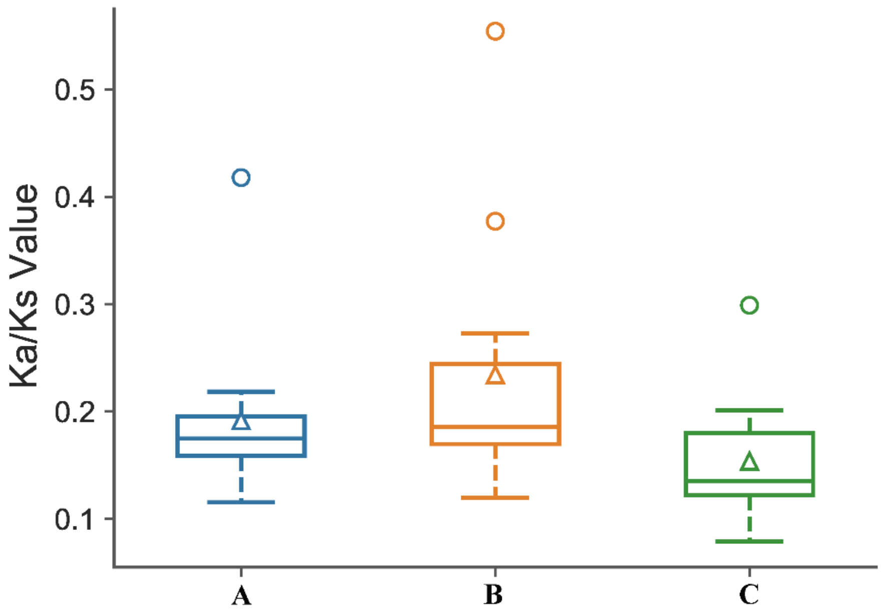

Figure S3: Distribution of Ka/Ks ratios of NOT2_3_5 gene pairs in tetraploid cotton.
A: Ka/Ks ratios between A and D sub-genomes of *G. hirsutum*. B: Ka/Ks ratios between *G. hirsutum* and *G. barbadense.* C: Ka/Ks ratios between A and D sub-genomes of *G. barbadense.*

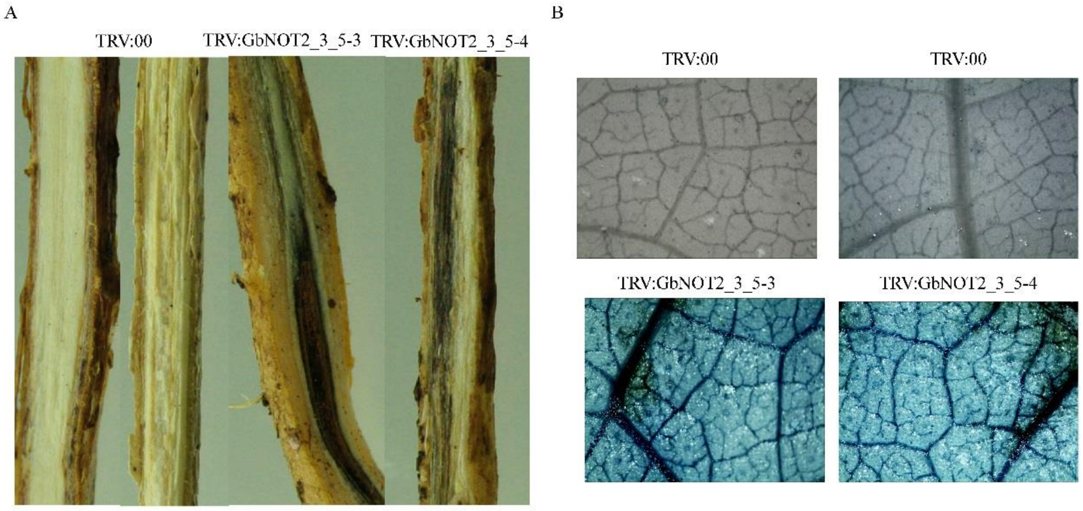

Figure S4: The stems and leaves of silencing *GbNOT2_3_5* genes plants showed more severe symptoms after infection of *V. dahlia*. A: The stems from silencing *GbNOT2_3_5* genes plants and control. B: Trypan blue array of leaves from silencing *GbNOT2_3_5* genes plants and control.
